# Elephantiasis Arabum of the Left Inferior Extremity Treated Successfully, by Ligature of the Femoral Artery

**Published:** 1858-02

**Authors:** J. M. Carnochan

**Affiliations:** Prof. of Surgery in the New York Medical College, Surgeon-in-Chief to the State Hospital


*Elephantiasis Arabum of the Left Inferior Extremity treated successfully, by Ligature of the Femoral Artery.* By J. M. CARNOCHAN, M.D., Prof. of Surgery in the New York Medical College, Surgeon-in-Chief to the State Hospital.

In September, 1852, I recorded a case of Elephantiasis Arabum successfully treated, by ligature of the femoral artery. Since then, I received a letter from Professor Erichsen, of the London University, in which he mentions a case of Elephantiasis treated, with a successful result, upon the same principle.

The portion of his letter referring to this subject runs as follows :— "I have perused the details of your case with great interest, and have been especially struck by the account of the successful ligature of the femoral artery for that otherwise intractable disease, Elephantiasis Arabum. The operation was certainly a bold step, but one that the result shows to have been the proper one to take ; and it certainly does infinite credit to your judgment and skill, to have devised a successful treatment for this complaint. I have at present under my care a man with extensive Elephantiasis of the foot, in whom, about two months ago, the Assistant Surgeon, during my absence, tied the anterior tibial, in the middle third of the leg. This case has done well, and the limb is fast recovering its usual dimensions. The operation was performed on hearing of the success of your case."

The following case is another example of the beneficial results of this practice, in a disease hitherto deemed incurable by other means :

Francisco Podesta, a native of Italy, 39 years of age, peasant by occupation, was admitted into the State Emigrant's Hospital, on the 17th of April, 1857. About six years previous to his admission, after prolonged exposure to cold and dampness, he was attacked with severe pains in the left leg, extending upwards along the thigh. The pain was followed by general tumefaction of the leg from the toes upward to the knee joint. The local disease was attended by constitutional disturbance, which, however, gradually subsided, the limb remaining much disfigured and considerably larger than that of the opposite side. The foot was thickened, and appeared stunted from the increased circumference around the ankle; the calf of the leg was also considerably augmented; the skin and subcutaneous tissues were dense, hard, and hypertrophied ; the surface of the limb below the knee was scaly, and, in general, presented the unseemly appearance from which the term Elephantiasis has been derived. Around the ankle the hypertrophied tissues were thrown into large and prominent folds, hanging, as it were, over the ankle joint ; between two of these folds an extensive ulceration existed, as large as a dollar piece. The patient, in this condition, was unable to follow any vocation ; in fact, was unable to walk, and entered the hospital more as an asylum, than with the hope of obtaining relief. His general condition was feeble, and he presented a cachectic appearance. The following were the measurements of the limb : the thighs at the middle were of equal measurement, 19 inches ; around the calves on the sound side, 10 inches ; on the diseased side, 13 inches ; above the ankles on the sound side, 8¼ inches ; on the diseased side, 12 inches ; around the ankles on the sound side, 9¼ inches ; on the opposite side, 15½ inches, while the measurement from the heel around the instep on the sound side was 12 inches, and 19 inches on the diseased side.

Deeming it unnecessary to repeat in this patient's case the numerous remedies which had been unavailingly tried, I proposed to apply a ligature upon the femoral artery, for the purpose of modifying the morbid nutrition of the limb. Allowing him some time to improve his general health, and to become accustomed to the air of the hospital, on the 23rd of May a ligature was applied on the femoral artery in Scarpa's space. The artery was found to be healthy and of normal size. May 27th: Everything had progressed favorably up to this time, and the limb was beginning to show evidence of diminution. July 1st, the ligature came away; at this time, the calf of the diseased side had diminished 1 inch above the ankle; around the ankle, 1¾ inches, and in the measurement in the heel around the instep, 3½ inches; the skin of the leg and foot was also soft. July 10th, the patient was allowed to move about, the ankle joint which previously was almost immovable, now admitted of flexion and extension. The ulceration had now healed, and from this time forward until the 24th of August, (the time of his discharge from the hospital,) the dense and hardened tissues were gradually becoming more natural and soft. He left the hospital with his limb almost of natural size, and able to walk with but slight lameness.

45 La Fayette Place, Dec. 10th, 1857.

[*American Medical Gazette.*

